# Impact of Drought and Biostimulant in Greenhouse Tomato: Agronomic and Metabolomic Insights

**DOI:** 10.3390/plants14132000

**Published:** 2025-06-30

**Authors:** Marzia Leporino, Mariateresa Cardarelli, Paolo Bonini, Simona Proietti, Stefano Moscatello, Giuseppe Colla

**Affiliations:** 1 Department of Agriculture and Forestry Science, University of Tuscia, 01100 Viterbo, Italy; marzia.leporino@unitus.it (M.L.); tcardare@unitus.it (M.C.); 2 OloBion SL, 08028 Barcelona, Spain; pb@olobion.ai; 3 National Research Council (CNR), 05010 Porano, Italy; simona.proietti@cnr.it (S.P.); stefano.moscatello@cnr.it (S.M.)

**Keywords:** water stress, *Solanum lycopersicum* L., plant-derived protein hydrolysate, antioxidants, fruit quality

## Abstract

Widespread drought conditions have increasingly affected agricultural productivity, requiring the exploration of alternative approaches for improving crop tolerance, yield and quality, since plants adopt many physiological strategies to cope with challenging environments. This study evaluated the effects of a vegetal-derived protein hydrolysate (PH), applied via foliar spray or root drench at a concentration of 3 mL L^−1^, on tomato plants (n = 96) under well-watered and drought-stressed conditions over a 136-day greenhouse experiment. Overall, sub-optimal irrigation significantly decreased plant dry biomass (−55.3%) and fruit production (−68.8% marketable yield), and enhanced fruit quality in terms of sugar concentration and antioxidant levels. PH treatments, regardless of the application method, did not notably influence above-ground dry biomass, yield, or fruit quality, suggesting that the intensity of drought might have limited PH effectiveness. Metabolomic analysis showed higher concentrations of stress- and quality-related metabolites in tomato fruits from plants under stress, with PH not exerting significant metabolic changes in the fruits. These findings revealed the diminished effectiveness of PHs under severe drought conditions, suggesting that drought stress level needs to be taken into consideration for optimizing biostimulant efficacy.

## 1. Introduction

Widespread climate change has worsened the frequency and extent of drought conditions, which are a cause of reduction in agricultural productivity worldwide [1]. Limited rainfall and high temperatures often occur during key plant growing stages, exacerbating water supply issues in many agricultural regions, where irrigation resources are frequently insufficient. This is the reason why optimizing water use through improved irrigation strategies has become essential to mitigate the effects of prolonged drought periods [2].

Plants primarily suffer drought stress when subjected to prolonged low soil moisture, triggering many physiological and biochemical responses aimed at reducing oxidative damage and preserving vital functions [3,4]. Water scarcity reduces plant water use efficiency, mainly by inducing stomatal closure to limit transpiration and prevent water loss, thereby altering photosynthetic efficiency and carbon metabolism, leading to a reduction in overall plant biomass [5]. Imbalances related to photosynthesis cause a downregulation of photosystem II (PSII), resulting in an accumulation of unused electrons that are converted into reactive oxygen species (ROS), potentially damaging cellular structures [6]. Furthermore, drought stress speeds up pigment degradation, especially that of chlorophyll [5], while other pigments such as carotenoids and antioxidants—including phenolic compounds and flavonoids—act as non-enzymatic defence mechanisms to cope with oxidative stress and enhance plant stress tolerance [7,8].

These alterations are particularly significant when they impact horticultural crops such as tomato (*Solanum lycopersicum* L.), which is one of the most economically important vegetables cultivated in Italy, reaching approximately 5.5 million tonnes per year [9]. Under limited water availability, morpho-physiological alterations in plant tissues can impair fruit development, by disrupting the transport of essential macro- and micronutrients to the fruit [10]. Furthermore, water shortage reduces cell growth and water content in fruit, thus reducing their size and weight, putting them at risk for physiological disorders such as blossom-end rot [11]. Conversely, fruit firmness can be enhanced by lower water content because it has a direct relation with increased dry matter accumulation and tissue density that influences the mechanical resistance of the fruit. Drought affects fruit composition by altering photosynthetic activity, sugar metabolism, and carbohydrate translocation, frequently resulting in increased sugars including glucose, fructose, and sucrose—which act as osmolytes—due to decreased water content of the fruit, enhancing sweetness, flavour, and promoting increased total soluble solids (TSS), a critical quality trait in tomato [12,13]. Aside from sugar accumulation, drought stress may also enhance the accumulation of antioxidant secondary metabolites including polyphenols, flavonoids, and carotenoid pigments such as lycopene. These compounds have a protective role against oxidative stress by stabilizing reactive oxygen species (ROS) in plants and are also recognized for their health-promoting effects in human nutrition [12,13].

Considering the necessity to balance water-use efficiency, yield, and fruit quality, sustainable alternatives such as biostimulants have gained attention for their potential to promote plant tolerance to drought stress. Among biostimulants, vegetal-derived protein hydrolysates (V-PHs) have been shown to be beneficial in enhancing crop performance, especially in suboptimal growing conditions, thanks to the bioactive peptides and amino acids which are made according to [14]. When applied to plants, these compounds trigger many molecular and physiological responses under water stress conditions. For example, in a tomato experiment [15], a plant-derived protein hydrolysate improved water status and pollen viability under drought conditions, resulting in six-fold higher yields compared to untreated plants. Furthermore, V-PH modulated antioxidants in both leaves and fruits, depending on the irrigation level used. Agliassa et al. [16] found that applying a V-PH to pepper plants (*Capsicum annum* L.) resulted in faster drought recovery, and a higher relative growth rate (+61.8%) and fruit yield (+64.7%), when compared to untreated stressed plants. Nevertheless, the efficacy of V-PHs in enhancing drought tolerance can vary as a function of drought severity and duration. In a short-term experiment on tomato, the application of a Malvaceae-derived PH led to faster stress recovery following repeated water stress events, likely due to stress-related metabolic reprogramming of plant cells. Although the PH did not significantly affect plant biomass, a trend was observed toward greater accumulation under stress conditions when the biostimulant was applied [17]. However, such findings must be validated in long-term experiments because the ultimate target is tomato production, and, under prolonged severe drought, the damage to plant metabolism and structure may be beyond a recovery point through biostimulants, resulting in significant yield loss. In terms of application methods, a short-term experiment considering only the vegetative phase of tomato plants demonstrated that root drenching with PH improved final biomass by 33% compared to foliar spraying with PH under water stress conditions [18]. However, the optimal timing and method of application—whether foliar spray or root drench—requires further investigation, particularly in the context of long-term experiments. These knowledge gaps need to be addressed for developing targeted recommendations that can maximize the benefits of biostimulants in commercial tomato production.

Given its demonstrated efficacy in a short-term experiment on tomato under water stress [17], a Malvaceae-derived protein hydrolysate (PH) was considered for this long-term study. Taking these aspects into consideration, this study aimed to assess the effect of the PH applied through foliar application or soil drenching on tomato plants grown under optimal and sub-optimal water availability conditions. We hypothesized that PH application would mitigate the negative effects of water stress on plant growth, yield, and fruit quality, and the mode of application (foliar vs. root) would influence the extent of drought stress.

## 2. Results

### 2.1. Growth Traits and Fruit Yield

Biometric measurements recorded at harvest on the dry weight (DW) of leaves, stems, fruits, and above-ground biomass (Supplementary Table S1) showed that only irrigation significantly influenced these traits, as stressed plants showed reduced DW of all plant components and total biomass than those well-watered. In stressed plants, DW decreased by 56.1% in leaves (96.0 vs. 149.9 g/plant), 49.8% in stems (55.2 vs. 82.7 g/plant), 58.3% in fruits (157.4 vs. 249.1 g/plant), and 57.2% in the above-ground biomass (308.1 vs. 484.3 g/plant) when compared to well-watered plants. The application of protein hydrolysate (PH) either foliarly (PH-F) or at the root level (PH-R) did not reveal a statistically significant effects on biomass accumulation when compared to the untreated plants (U) (avg. DW g/plant: leaves 123.51, stem 68.83, fruits 203.33, and above-ground biomass 396.36), and no significant interactions between irrigation and biostimulant treatments were recorded.

Concerning plant yield (Table 1), stressed plants exhibited significantly lower fruit production compared to the well-watered ones, as shown in Table 1: −68.8% for marketable yield, −67.5% for total yield, −47.1% of number of marketable fruits, and −14.0% for fruit weight. Unmarketable fruits, which included rotten and underdeveloped fruits, observed a decrease of 43.3% in stressed plants. The PH treatments and its interaction with irrigation had no significant impact on fruit yield and yield components. By averaging the results of yield traits for treatments, marketable yield reached 2147.36 g/plant, with a total yield of 2467.19 g/plant, number of marketable fruits of 129.10 n./plant, fruit weight of 16.27 g/fruit, and not-marketable yield of 319.83 g/plant.

### 2.2. Leaf Non-Destructive and Destructive Pigment Measurements

Except at 20 days after transplanting (DAT), the chlorophyll index (Supplementary Table S2) was always significantly modulated by the irrigation level, with higher chlorophyll content in stressed plants compared to well-watered plants. The interaction between irrigation and biostimulants was significant at 20, 55, and 133 DAT. Watered plants (W) treated with PH-R showed greater chlorophyll levels at 20 DAT, whereas in the other two cases (55 and 133 DAT), higher values were observed in stressed plants (S). At 55 DAT, the highest chlorophyll content was found in untreated (U) stressed plants (S × U, 0.906), followed by S × PH-R, W × U, W × PH-F, and S × PH-F (0.904, 0.861, 0.857, and 0.849, respectively). W × PH-R-treated plants exhibited the lowest values (0.793). The situation evolved in the following measurements, showing a tendency for increased chlorophyll index in PH-treated plants under stress, especially with significantly higher chlorophyll for S*PH-F-treated plants at 133 DAT (1.253 vs. 1.140 and 1.129 for S × U and S × PH-R, respectively), whereas watered treatments consistently exhibited lower values.

The mid-crop cycle pigment assessment of chlorophyll a, chlorophyll b, total chlorophyll, and carotenoid content revealed significantly higher values for all pigments in stressed plants compared to well-watered plants (Table 2). Among biostimulant treatments, plants treated with PH-R exhibited significantly increased levels of chlorophyll a, chlorophyll b, and total chlorophyll compared to PH-F, while carotenoid values remained consistent among treatments. When combined with irrigation levels, PH-R significantly altered chlorophyll, particularly chlorophyll a and total chlorophyll in stressed plants, resulting in the highest pigment concentration. A similar pattern was observed in chlorophyll b and carotenoids, although they were not significantly affected from biostimulant treatments.

### 2.3. Fruit Quality 

Table 3 shows statistical analysis for fruit quality parameters (°Brix, pH, titratable acidity, dry matter, and firmness assessed at the IV truss). Irrigation had a significant impact on all measured fruit quality indicators, except for TSS/TA, while PH application and its interaction with irrigation showed no significant effects. Fruits grown under water stress (S) showed higher °Brix, titratable acidity, dry matter content, and firmness than fruits grown under well-watered conditions (W). The only parameter with the opposite trend was pH, which had a lower value under water stress conditions.

The diameter of tomato fruits, measured on marketable fruits from the first to the last truss (I–VIII) (Supplementary Table S3), was significantly higher in watered plants than in stressed plants. The results for the biostimulant factor revealed that the PH had a significant effect on the fruit diameter only on the IV truss, with bigger fruits in plants treated with PH-R. The interaction between irrigation and biostimulant was not significant for fruit diameter.

### 2.4. Fruit Nutrient Composition

Potassium (K), among macronutrients, and micronutrients (manganese (Mn), zinc (Zn), and copper (Cu)) were significantly affected by irrigation (Table 4). The nitrogen (N) and phosphorus (P) concentrations remained unaffected by irrigation. Fruits under well-watered conditions had higher K (50.64 g/kg DW), Mn (62.74 mg/kg DW), Zn (25.44 mg/kg DW), and Cu (5.63 mg/kg DW) compared to those grown under stress conditions, which showed reduced levels in all nutrients mentioned. The application of the protein hydrolysate (PH) significantly affected only iron (Fe), reaching the highest levels in the foliar treatment (PH-F, 325.85 mg/kg DW), while the untreated control (U) showed the lowest value (250.01 mg/kg DW); PH applied as root drench (PH-R) provided intermediate Fe concentration (303.05 mg/kg DW) among biostimulant treatments. No statistical differences were observed in the interactions between irrigation and biostimulant treatments for any of the nutrients analysed.

### 2.5. Fruit Antioxidants

The antioxidant capacity of tomato fruits was determined at mid-crop cycle by assessing the Ferric Reducing Antioxidant Power (FRAP), 2,2-diphenyl-1-picrylhydrazyl (DPPH), as well as polyphenol and flavonoid concentrations (Table 5).

FRAP was significantly altered both by irrigation and biostimulant treatments, but not by their interaction. FRAP was increased in stressed plants compared to well-watered plants (+14.7%), while in the case of PH treatments, foliarly-treated plants (PH-F) resulted in highest FRAP value. In contrast, neither of the main factors (irrigation, biostimulant) or their interaction had a significant effect on DPPH.

Polyphenols and flavonoids were significantly affected by irrigation level, showing higher values in stressed plants. In stressed plants, polyphenols concentration in fruits increased by 6.6% and flavonoids by 17.1% when compared to well-watered plants.

Neither irrigation nor biostimulant treatment significantly altered the lycopene content, with an average value of 31.6 mg/100 g FW.

### 2.6. Fruit Carbohydrates

Table 6 reports the fruit concentration of soluble carbohydrates (glucose, sucrose, fructose, and their sum) and starch. Among soluble sugars, only glucose and sucrose were significantly affected by irrigation, showing different trends: glucose increased in stressed plants compared to well-watered plants (+13.9%); conversely, sucrose decreased (−34.8%). Protein hydrolysate (PH) treatments combined with irrigations levels modified glucose content, with stressed plants (S) treated with root drench (S × PH-R) showing an increase in comparison with the other combinations. Specifically, watered untreated plants (W × U) or plants treated with PH-R, S × U, and S × PH-F exhibited intermediate behaviour between S × PH-R (296.21 mg/g DW) and W × PH-F (208.40 mg/g DW), which had the highest and lowest glucose values. The average starch concentration was 5.3 mg/g DW.

### 2.7. Fruit Metabolomics

Metabolomics analysis was performed on marketable fruits of different treatment combinations. The analysis revealed the modulation of 278 metabolites associated with pathways involved in the development of stress resilience and fruit quality.

**Figure 1:**
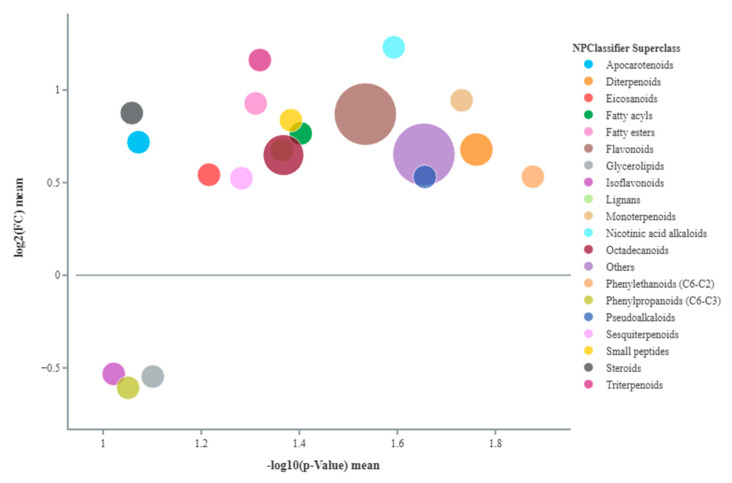
Comparison between drought stressed and well-watered tomato fruits under no biostimulant application for metabolite classes identified through chemical enrichment. The figure displays the log2(FC) mean and the -log10(*p*-value) mean of the metabolites grouped by class. Each dot’s size represents the sum of the fold change (FC) of the class. A log2(FC) mean greater than 0 indicates upregulation of the class, while a value lower than 0 indicates downregulation. Moving to the right on the *x*-axis, the -log10(*p*-value) mean corresponds to a higher significance in metabolite changes.

A comparison was made between untreated plants under sub-optimal irrigation and untreated well-watered plants. In Figure 1 the main metabolite classes identified in marketable fruits are shown. Amino acids such as L-proline, L-2,3-dihydrodipicolinate, and DL-arginine increased under stress, particularly proline, with a fold change (FC) of 1.79. Many pigments and their derivatives were also modified under stress conditions. Anthocyanins such as cyanidin 3-O-(6-O-β-D-glucosyl-2-O-β-D-xylosyl-β-D-galactoside) and apocarotenoids like 5,6-epoxy-3-hydroxy-β-ionone and β-D-glucopyranosyl abscisate (FC 1.21, 1.65, and 1.20) were significantly upregulated. Stress also increased flavonoids and flavonoid-related compounds. For example, chalcones like phlorizin, butein, and 3′,5′-di-C-glucosylphloretin increased. Dihydroflavonols, such as dihydromyricetin, and flavonones, such as hesperetin-7-O-β-D-glucoside and naringin, were also upregulated. In terms of phenolic compounds, cinnamic acids and derivatives such as 1-O-feruloylglucose and 1-O-cinnamoylglucose increased significantly. Many gibberellins (A24, A34, and methyl gibberellin A9) were more abundant in stressed plants, as was jasmonic acid. Other compounds like serotonin (an indole alkaloid), phenethylamine, and 2-phenylethyl β-D-glucopyranoside (among benzene derivatives and organooxygen compounds, respectively) increased, while wax monoesters such as 2,3-dioctanoylglycerylceramide decreased.

When the metabolites of fruits of plants treated with PH both foliarly (PH-F) or as root drench (PH-R) are compared to untreated stressed fruits, no significant differences emerged in their metabolic profiles, as well as when watered fruits treated with PH-F or PH-R were compared to untreated watered fruits.

## 3. Discussion

In the current trial, irrigation was the most important factor influencing plant biomass accumulation, both in terms of vegetal biomass and fruit production, with stressed plants producing lower dry weights of leaves, stems, fruits, and total above-ground biomass than well-watered plants. Overall, the decrease in dry weight across all parameters in stressed plants confirmed that the plant growth was strongly dependent on adequate water supply. Furthermore, there was no significant difference between the biomass accumulation of the plants treated with the protein hydrolysate (PH), applied either foliarly (PH-F) or as root drench (PH-R), and the untreated control (U), suggesting that water stress severely impaired plant metabolism, limiting the PH-mediated stress mitigation activity. Similarly, Patanè et al. [19] demonstrated a significant drop in total dry biomass in tomato plants (−56.3%) when a severe water deficit was imposed immediately following plant establishment, as in the current experiment, whereas the results obtained with a 50% reduced water rate from early stages or from flowering were comparable to 100% watered plants. These findings were also reflected in fruit yields.

Tomato yield showed a similar trend of plant biomass accumulation, clearly supporting the dominant effect of irrigation level on crop growth and productivity. All yield parameters were significantly reduced under water stress conditions, with an approximate 70% decrease in both marketable yield and total yield, indicating the importance of adequate water supply for fruit productivity. The halving of the number of marketable fruits indicated a limited fruit set; additionally, while the average fruit weight decreased moderately, this was consistent with the observed improvement in fruit size and, as a result, higher fruit marketability under well-watered conditions. These outcomes suggest that, in addition to the imposed water stress, the limited effectiveness of the protein hydrolysate (PH) used—previously shown to promote recovery under short-term drought [17]—may also be attributed to suboptimal dosage and timing of application, possibly constrained by stomatal closure induced by stress conditions. In the present experiment, the expected improvements in tomato yield due to PH application were not observed. Even under well-watered conditions, PH did not significantly enhance yield or biomass, serving primarily as a positive control, with the aim of assessing the PHs’ efficacy under drought stress conditions. In water-stressed plants, the lack of PH efficacy may reflect a combination of factors, including the severity, timing, and duration of stress. As stress was imposed from early developmental stages and continued over the entire cycle, the plants’ physiological capacity to respond to external treatments may have been compromised. Although vegetal protein hydrolysates are well-documented for improving drought tolerance, the extreme conditions applied in this study likely exceeded the threshold of PH effectiveness. Indeed, previous studies have shown that biostimulants derived from vegetal sources can help plants to mitigate the negative effects of drought. For example, protein hydrolysate-based biostimulants improved tomato yield under limited water availability (50% irrigation compared to 100% irrigation) [15], and seaweed extract from *Ascophyllum nodosum* applied at 50% irrigation also enhanced tomato crop performance (higher yields when compared to untreated plants) under reduced water conditions [20]. In the present experiment, soil moisture recorded by in-pot sensors showed that water-stressed plants reached several times the temporary wilting point (30% probe reading). In these conditions, root water uptake is impaired, as well as stomatal closure, leading to reduced photosynthetic activity and carbohydrate availability, which represent key factors for biostimulant signal transduction and metabolic activation. Additionally, extended stress may have impaired transport mechanisms, limiting the uptake and effectiveness of PH compounds, suggesting that biostimulants are effective when applied under moderate, well-timed stress conditions. Undeniably, under extreme drought conditions, plant functions like photosynthesis and transpiration may be seriously hindered [21], consequently affecting the plant’s ability to respond to biostimulant signals or properly uptake the bioactive compounds and distribute them for an effective use.

The dynamics of chlorophyll content measured non-destructively throughout the tomato crop cycle provided valuable insight into the physiological responses to both water availability and biostimulant treatments. At 20 days after transplanting (DAT), the significant interaction between irrigation and PH treatments had limited physiological relevance, as the effects of only two PH applications can be considered minimal. The higher chlorophyll content in watered plants treated with PH-R at this stage may have simply reflected initial variability rather than a meaningful biostimulant impact. At 55 DAT, the observed increase in chlorophyll concentration in stressed plants could be attributed to both a compensatory response and to higher concentration in smaller leaves, as demonstrated by Rosa et al. [22], who studied changes in tomato leaf reflectance associated with chlorophyll during water scarcity. Reduced leaf expansion is a common drought response, likely resulting in denser packing of chlorophyll within smaller and thinner leaves, as also evidenced by the significant reduction in leaf biomass under water stress. Thus, the higher chlorophyll readings in water-stressed plants are likely a result of a morphological adaptation rather than an active improvement of pigment biosynthesis. However, PH-F-treated stressed plants showed encouraging results, suggesting that the PH-F may have started enhancing stress tolerance mechanisms at a time when water stress had not yet imposed irreversible damage to the photosynthetic system, helping to preserve chlorophyll and reduce oxidative degradation. By the end of the trial (133 DAT), the shift to lower chlorophyll content in PH-R treated plants under stress in comparison with PH-F—which resulted in the highest chlorophyll content—suggests that foliar-applied PH may have initially supported photosynthetic pigment preservation, but was ultimately unable to prevent stress-induced damage as drought became more severe and prolonged, in favour of a slightly better response in plant treated with root drench. Destructive measurements of chlorophyll a, chlorophyll b, total chlorophyll, and carotenoids in samples harvested at the mid-crop cycle validated non-destructive measurements made during the same period. Pigments increased significantly in water-stressed plants [23], possibly due to their higher concentrations compared to watered and more developed plants. Furthermore, among biostimulants, PH-R significantly increased levels of chlorophyll a and total chlorophyll, whereas PH-F showed lower chlorophyll content under stress, closer to levels found in watered plants. This could indicate that PH-F treated plants had a less pronounced stress response and better stress mitigation. The unchanged values in carotenoid concentration suggest a selective modulation of the photosynthetic system, possibly reflecting a targeted action of PH-derived peptides and amino acids on chlorophyll stabilization. Nevertheless, these moderate differences in chlorophyll dynamics did not translate into significant improvements in biomass accumulation or yield, further supporting that under severe and extended water stress, neither application method of PH provided a strong enough protective effect to maintain productivity.

In terms of fruit quality traits, fruits grown under water stress exhibited higher °Brix values (8.83), suggesting greater soluble sugar content due to the reduced water uptake and smaller fruit size, which typically results in enhanced fruit sweetness and flavour. Similarly, the increase in titratable acidity (0.48 vs. 0.46 g citric acid 100 mL^−1^ juice) in stressed fruits likely corresponded to a greater presence of organic acids, which, combined with the higher °Brix, can further improve the taste of the fruits. Despite the lack of significant differences among treatments, TSS/TA results are in line with high quality tomatoes in terms of flavour and ripening stage [24]. The lower pH in stressed fruits (4.29 vs. 4.32) was also consistent with the higher acidity, implying a higher concentration of acidic metabolites. Dry matter content, which was significantly higher under drought (9.72% vs. 9.30%), indicates a higher proportion of solids in relation to water, often associated with improved nutritional value and extended shelf life. Firmness, which is critical for transportation and shelf-life, was also higher in drought-stressed fruits (1.09 N vs. 0.95 N), likely due to reduced cell expansion and water content, which increase tissue density and resistance to deformation. These results are consistent with many studies on tomatoes, where improved quality characteristics were observed when subjected to water shortage [11,25,26]. Fruit diameter observed an isolated increase for PH-R treated plants, suggesting a potential transient benefit of root drenching. However, this effect was not sustained across trusses. The lack of significant interaction between irrigation and PH treatments supports that PH applications were not sufficient to modify fruit quality outcomes under the imposed stress conditions. Overall, while drought stress reduced fruit size, it enhanced several quality traits such as sugar content, acidity, and firmness—parameters that are particularly relevant for the cherry tomato market value and consumer preferences.

On the other hand, drought stress negatively affected the nutrient composition of tomato fruits, particularly potassium (K), manganese (Mn), zinc (Zn), and copper (Cu), which were all significantly reduced under limited water availability, potentially impacting the nutritional value of tomato fruits as source of micronutrients for human diet [27]. This decline could be attributed to the constant low soil moisture during the crop cycle and reduced transpiration rates that may have impaired nutrient mobility and root structure and uptake, as well as compromised internal transport within the plant and to the fruits. For example, Mitchell et al. [28] discovered that tomato fruit K levels were significantly reduced in response to soil water deficit, affecting sugar balance in sink organs rather than net carbon accumulation, while reduced Mn, Zn, and Cu concentrations may relate more to lower yields in stressed plants that likely influenced their fruit concentrations as well [29]. This micronutrient imbalance towards lower values may have affected Fe concentration [30], which was significantly increased by the application of PH-F in comparison with untreated or root-drenched plants (PH-R). This suggests that while water deficit can disrupt the assimilation of many essential nutrients, specific biostimulant strategies (e.g., molecular signaling) may have bypassed root-related limitations, promoting direct Fe assimilation or remobilization by the leaves and old tissues, facilitating systemic translocation to developing fruits, which function as plant sinks.

The results demonstrated that drought stress significantly enhanced the antioxidant potential of tomato fruits, particularly as measured by FRAP, polyphenol, and flavonoid content. The increased FRAP values in stressed plants suggest an upregulation of antioxidant defence mechanisms in response to oxidative stress caused by limited water availability. FRAP was also significantly stimulated in plants treated with PH-F, indicating that foliar application may have further increased antioxidant capacity, possibly by promoting the synthesis of non-enzymatic antioxidants or enhancing redox homeostasis [15]. The increase in total polyphenols and flavonoids in fruits from stressed plants is in line with the higher FRAP values and reflects a typical biochemical response to the water deficit [13]. These secondary metabolites are directly involved in tomato fruit nutritional and functional quality; therefore, their enhancement under water stress suggests that, despite a yield reduction, fruit quality in terms of antioxidants richness can be improved.

The carbohydrate profile of tomato fruits under water stress revealed a metabolic adjustment for enhancing osmotic regulation and stress tolerance. The higher accumulation of glucose and fructose in comparison with sucrose content suggests an active conversion of sucrose in its constituents (glucose and fructose) through enzymatic processes. Despite the lack of enzymatic activity analysis, this shift is most likely due to the activation of invertase enzymes, which are known to be upregulated during drought conditions to facilitate sucrose hydrolysis [31]. This mechanism plays a crucial role in maintaining fruit cellular turgor and protecting cells from dehydration [32]. In particular, when observing the interaction between irrigation and PH, PH-R under stress recorded the higher values of glucose, suggesting that root drenching may further stimulate sugar metabolism, potentially influencing the expression of carbohydrate-related enzymes and enhancing the adaptive response under stress. Although fructose was not significantly affected by treatments or their combination with irrigation levels, it remained the predominant sugar, supporting the typical sweetness profile of cherry tomato. Still, the increased levels of glucose and fructose under drought conditions may have enhanced the organoleptic quality of tomato fruits, despite the decreased yield observed in this experiment; this also indicates an adaptive response that supports the production of health-promoting antioxidants, thereby positively affecting overall fruit quality.

In terms of metabolic profiling, the comparison of untreated stressed fruits and well-watered fruits revealed the accumulation of key metabolite classes in the plants’ adaptive response to drought and fruit quality. For instance, the increased levels of amino acids in stressed fruits suggest the activation of osmotic adjustments, reactive oxygen species (ROS) detoxification, and nitrogen storage, aiding to maintain the integrity of proteins and membranes during dehydration, while also serving as signaling molecules [33,34]. Among amino acids, proline notably increased, likely playing a role in stabilizing cell membranes and proteins, while also functioning as an antioxidant and signaling molecule that offers additional defence against water loss [35]. L-2,3-dihydrodipicolinate was found to participates in lysine biosynthesis, which is another compound directly involved in plant stress response and development [36]. L-arginine, one of the most versatile amino acids, was probably involved in maintaining fruit cell functions [37] or acted as a precursor of compounds such as polyamines, which actively contribute to fruit development and ripening by indirectly affecting firmness, sugar metabolism, and antioxidant capacity [38]. Notably, whereas anthocyanins such as cyanidin 3-O-(6-O-β-D-glucosyl-2-O-β-D-xylosyl-β-D-galactoside) may play a role in mechanisms of stress tolerance [39], apocarotenoids like 5,6-epoxy-3-hydroxy-β-ionone and β-D-glucopyranosyl abscisate are more closely associated with the flavour and aroma of tomato fruits, in addition to acting as signal molecules in stress responses [40,41]. The physiological function of phlorizin, classified as a dihydrochalcone, remains not completely understood; however, it is acknowledged for its positive impact on human health [42]. Interestingly, the increase in butein could be linked not only to higher cuticular wax production, which improves fruit protection, but also because, as a flavonoid-related compound, it may represent a beneficial phytochemical substance [43]. Upregulation of dihydromyricetin (a dihydroflavonol), hesperetin-7-O-β-D-glucoside, and naringin (flavonones) may indicate wider flavonoid pathway activation. 1-O-feruloylglucose and 1-O-cinnamoylglucose are glycoside derivatives of ferulic and cinnamic acids, and as part of the larger class of polyphenols [44]—together with flavonoid derivatives—further corroborate the nutritional value of tomato fruits, which may resulted improved under water deficit [45]. Other compounds possibly contributing to tomato fruit quality included serotonin, acknowledged as a beneficial compound for human health and found to accumulate particularly in fresh tomatoes [46]; vitamin C, which was also reported to increase under water stress by Conti et al. [47], is known for its beneficial role in human nutrition, while phenethylamine and 2-phenylethyl β-D-glucopyranoside play a key role in flavour volatile synthesis [48]. Conversely, the reduction of 2,3-dioctanoylglycerylceramide, a type of wax monoester, might just be linked to a transition to alternative protective mechanisms in the fruits. Some gibberellins (A23 and A34) were found to be increased, even though their reduction is usually associated with a rapid response to water stress [49], and it could be associated to the regulation of fruit ripening [50] and homeostasis with methylated forms (methyl gibberellin A9), which are known to play a direct role in stress tolerance [51], along with jasmonic acid [52]. In summary, all these metabolites highlight the potential of taking advantage of water stress to improve nutritional and organoleptic traits of tomato fruits. However, the lack of significant differences in the metabolite profiles of tomato fruits treated with PH—regardless of the application method—and untreated plants, both under well-watered and stressful conditions, suggest that the PH treatments did not induce measurable metabolic changes in the fruit tissue at the time of analysis. This could relate to compromised root function, which altered PH-R’s potential efficacy related to overall plant stress response. A possible explanation could also be that the sampling time did not coincide with the peak of PH-induced metabolic responses. Overall, these data indicate that while PHs may change plant metabolism, the direct effects on tomato fruit biochemistry were limited. This result reinforces the notion that sampling time and stress intensity are important factors to consider when trying to interpret the application of biostimulants, including potential changes in metabolomic analysis.

## 4. Materials and Methods

### 4.1. Plant Materials and Growth Conditions

**Figure 2:**
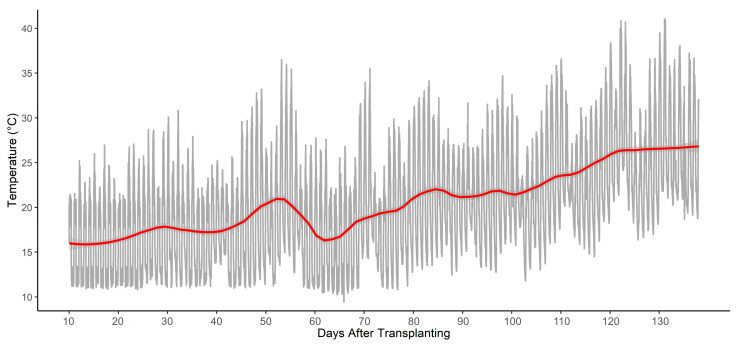
Temperature (°C) measured during the crop cycle. The grey line represents the raw temperature readings, while the red line illustrates a smoothed trend calculated as LOESS (Locally Estimated Scatterplot Smoothing) by using local regression on the daily temperature recordings.

The experiment took place in a polymethyl greenhouse at the University of Tuscia’s Experimental Farm (Viterbo, Italy, 42°25′ N; 12°08′ E; 310 m a.s.l.) from 20 February to 5 July 2024. During the first month, a heating system was used to keep the temperature above 20 °C at night, reducing cold stress after transplantation, while a ventilation system and side openings were automatically activated to regulate the daily air temperature when it exceeded 27 °C. A sensor from Toro Company (Tempus Air MS, Bloomington, MN, USA) recorded temperature trends throughout the trial, as shown in Figure 2.

Tomato seeds (*Solanum lycopersicum* L.—cv Pralyna, SAIS Sementi, Cesena, Italy) were sown in trays (170 holes/tray) filled with a commercial substrate containing primarily peat moss (Brill, Georgsdorf, Germany) and grown in a glass greenhouse at 25 °C with regular watering until the second true leaf reached full expansion. After one week, the seedlings were fertigated with NPK (20% N—8.8% P—16.6% K) at a rate of 1 g L^−1^. When the plants had two fully expanded leaves, they were transplanted into 9 L pots filled with sandy loam soil (70% sand, 30% soil). Ninety-six plants were distributed using a randomized complete block design, with 16 biological replicates per treatment (six treatments in total), and were pruned at the eighth truss stage. The pots were arranged in double rows, two for the stressed sector and two for the well-watered sector, with a plant density of 3.5 plant/m^2^.

Water soil sensors (Tempus Air MS, Toro Company, Bloomington, MN, USA) were placed in pots corresponding to each irrigation sector and connected to a remote platform. The substrate’s water content reading ranged from 50 to 80% in the well-watered sector and from 30 to 50% in the water-stressed sector. A probe reading of 80% corresponded to water container capacity, whereas a 30% probe reading corresponded to the temporary wilting point.

The nutrient solution (EC of 2.0 dS m^−1^ and a pH of 6.0), supplied through a drip irrigation system with 4 L h^−1^ emitters and managed through the sensors system, consisted of N-NO_3_ (10 mM), P (1 m M), S (1.25 mM), K (3.5 mM), Ca (3.5 mM), Mg (1.0 mM), Fe (17.1 μM), Mn (17.4 μM), Zn (3.7 μM), B (11 μM), Cu (1.9 μM), Mo (0.5 μM).

### 4.2. Biostimulant Application

A Malvaceae-derived protein hydrolysate (PH) was applied to plants either foliarly (PH-F) or at the root level (PH-R), and it was compared to an untreated control in both well-irrigated and stressed sectors. The PH contained 16.9% carbon and 4.67% nitrogen as free amino acids and peptides, as reported in the aminogram by El-Nakhel et al. [53]. Regarding foliar applications, PH was applied by uniformly spraying the leaves until complete wetting, using a diluted solution at a concentration of 3 mL L^−1^. For the root treatment, 100 mL of the same solution was applied directly to the soil in each pot. Control plants received distilled water applied in the same way and volume as those receiving the biostimulant treatments, ensuring consistent solution volumes across all treatments. Treatments were given every 10 days, starting 20 days after transplanting (DAT) and continuing until the end of the trial.

### 4.3. Fruit Yields and Yield Component

Tomato fruits were harvested on all plants between 27 May (97 DAT) and 3 July (134 DAT) due to scalar ripening of the tomato trusses. The yield was measured as total yield, marketable yield, number and average weight of marketable fruits, and not-marketable yield (rot and underdeveloped fruits). At the end of the trial, the harvested fresh biomass (leaves and stems) was recorded and oven-dried at 65 °C until it reached a constant weight for dry biomass measurement.

### 4.4. Leaf Non-Destructive and Destructive Pigments Measurements

Throughout the crop cycle, chlorophyll was non-destructively measured using a multi-pigment meter (MPM-100, ADC BioScientific Ltd., Hoddesdon, UK), which has an integrated clip that emits a flashing light to record pigment values as absolute units.

At mid-crop cycle and immediately before pruning (86 DAT), the fourth leaf from 8 plants per treatment was sampled, frozen in liquid nitrogen, and stored at −80 °C to determine total chlorophyll, chlorophyll a and b, and carotenoids, as described by Wellburn [54]. A quantity of 0.1 g of frozen leaf samples was extracted in acetone 80% (1:200 p/v) and homogenised using a homogenizer (IKA T10 basic UltraTurrax, IKA-Werke GmbH & Co. KG, Staufen, Germany). The extracts were centrifuged at 4800× *g* for 20 min. A spectrophotometer (Helios β, Thermo Fisher Scientific, Waltham, MA, USA) measured the absorbance solution at 663, 647, and 470 nm to determine the concentration of chlorophyll a and b, and carotenoids expressed as mg g^−1^ of fresh weight (FW). Total chlorophyll was calculated as the sum of chlorophyll a and b.

### 4.5. Fruit Quality Assessment

Subsamples of marketable fruits of the fourth truss were selected from each treatment to evaluate fruit quality parameters. Fruit firmness was measured on the equatorial zone of three fruits per plant (8 plants/treatment) using an automatic force transducer set to 3 N for tomato fruits (Instron, Pianezza, Torino, Italy), obtaining values expressed as newtons (N). On the same fruits, dry matter (DM) content was determined by oven-drying the samples at 65 °C until constant weight. Centrifuged and filtered subsamples (16 analytical replicates/treatment) were used for the determination of the soluble solid content (°Brix), pH, and titratable acidity (TA). °Brix were assessed on the filtered juice using a digital refractometer (Atago N1, Atago Co. Ltd., Tokyo, Japan). The pH was measured with a digital pH meter (HI-9023, Hanna Instruments, Padova, Italy); then, the juice was titrated with Stadtman et al.’s method [55] using 0.1 N NaOH to reach pH 8.1.

### 4.6. Fruit Nutrient Composition

Dried fruit samples (8 biological replicates/treatment) of the third truss were used for the total nitrogen determination according to the Kjeldahl method [56]. A quantity of 0.25 g of each treatment replicate was digested in 10 mL of 96% sulfuric acid, adding Kjeldahl catalyst tablet in Pyrex tubes. The digestion was carried out for 30 min at 420 °C in a digestor (DK6 Velp Scientifica, Usmate Velate, Italy). The distillation was then performed using the Kjeltec 2100 (Foss Analytics), adding 50 mL of 40% NaOH. Titration was conducted with 0.1 N HCl, using methyl red + bromocresol green as the indicator, and the titration volume was used to calculate the nitrogen percentage, which was then converted to g kg^−1^ of dry weight (DW). Dried samples were also used for the elemental analysis process. Six replicates from each treatment were placed in tubes along with 65% HNO_3_ and MilliQ water, then digested for 40 min (Multiwave GO Plus, Anton Paar GmbH, Rivoli, Torino, Italy). Following digestion, the samples were thinned with 1% HNO_3_ and examined using an atomic emission spectrometer (4210 MP-AES, Agilent Technologies, Santa Clara, CA, USA) connected with an SPS 4 Autosampler (Agilent Technologies, Santa Clara, CA, USA). A standard sample was always prepared as a reference for the elemental analysis.

### 4.7. Fruit Antioxidants Assesment

Subsamples of tomato fruits were collected (8 biological replicates/treatment), immediately frozen in liquid nitrogen and stored at −80 °C to determine antioxidant activity through Ferric Reducing Antioxidant Power (FRAP), 2,2-diphenyl-1-picrylhydrazyl (DPPH) and polyphenol and flavonoid assessment. The antioxidant capacity through FRAP and DPPH assays was evaluated through spectrophotometric measurements (Helios β, Thermo Fisher Scientific, Waltham, MA, USA) extracting 0.5 g of fresh tomato samples with 5 mL of 80% methanol homogenizing with UltraTurrax (IKA T10 basic UltraTurrax, IKA-Werke GmbH & Co. KG, Staufen, Germany).

The FRAP determination [57] was adapted and consisted in the incubation of 30 μL of tomato extract with 900 μL of FRAP working solution (0.3 M acetate buffer pH 3.6, 10 mM 2,4,6-tris(2-pyridyl)-s-triazine (TPTZ) in 40 mM HCl, 20 mM FeCl_3_ in distilled water) at 37 °C for 30 min. The samples’ absorbance was read at a wavelength of 593 nm based on the reduction of the ferric salt (Fe^3+^-TPTZ (2,4,6-tripyridyl-s-triazine)) to ferrous (Fe^2+^-TPTZ) under acidic conditions. The DPPH determination [58] consisted in the incubation of 100 μL of extract with 800 μL of 75 mM DPPH (15 mg in 50 mL 80% methanol) at room temperature for 30 min, and reading the extracts’ absorbance at 517 nm. For both methods, the antioxidant activity was expressed as mg Trolox equivalents (TE) g^−1^ FW.

The extraction for polyphenols and flavonoids was carried out on 0.5 g of each sample in 80% ethanol for spectrophotometric measurements. The polyphenol method ([59] with modifications) involved the incubation of 200 μL of extract with 1 mL Folin and Ciocalteau’s reagent (1:5), 800 μL of 7.5% Na_2_CO_3_ at room temperature for 1 h. The absorbance was recorded at 765 nm. For flavonoid quantification [60], 700 μL of extract was mixed with 150 μL of 5% NaNO_2_, 150 μL of 10% AlCl_3_, and 1 mL of 1 M NaOH. Samples were centrifuged due to the formation of colloids in suspension. The supernatant was read at 510 nm. In the case of polyphenols and flavonoids, the results were expressed as mg of gallic acid equivalents (GAE) g^−1^ FW and mg of quercetin equivalents (QE) g^−1^ FW, respectively.

Lycopene determination was performed as described by Adsule and Dan [61], with modifications, on 8 mg of freeze-dried samples of each treatment ground with mortar and pestle and mixed with 2 mL 100% acetone. Samples were incubated at room temperature for 30 min and centrifuged and diluted at 1:5; then, their absorbance was read at 505 nm with a spectrophotometer (Beckman DU-50 UV-visible; Beckman Instruments, Inc., Fullerton, CA, USA). Lycopene molar extinction coefficient was used to calculate samples’ lycopene content, expressed as mg 100 g^−1^ FW.

### 4.8. Fruit Carbohydrates Composition

Non-structural carbohydrates (NSC) were measured on 6 biological replicates/treatment using 10 mg of freeze-dried sample powder extracted in 50% ethanol maintained at 80 °C for 45 min under continuous shaking. After centrifugation, soluble sugars (glucose, fructose, and sucrose) were recovered in the supernatant, and starch remained in the pellet. The pellet was washed four times with 50 mM Na acetate buffer (pH 4.5), suspended and autoclaved at 120 °C for 45 min in 1 mL of the same buffer. Samples were then incubated at 50 °C for 1 h with amyloglucosidase (70 U) and α-amylase (4 U) to hydrolyze the starch to glucose. A spectrophotometric coupled enzymatic assay measured the glucose produced by starch hydrolysis. The supernatant containing soluble sugars was filtered through a nylon 0.2 µm PPII syringe filter (Whatman Inc., Maidstone, UK), then analyzed through high-performance anion exchange chromatography, with pulsed amperometric detection (HPAEC-PAD), using an ICS-6000 with a dual-eluent generator cartridge (EGC) module and an analytical CarboPac™ PA200 IC column for Dual EGC Mode (all equipment was ThermoFisher Scientific Dionex™, Waltham, MA, USA). Sucrose, glucose, and fructose were quantified against a carbohydrate standard curve prepared using HPLC-grade reagents (Sigma, Steinheim, Germany).

### 4.9. Fruit Metabolomics

Subsamples of tomato fruits (6 biological replicates/treatment) at the third truss stage were frozen in liquid nitrogen and kept at −80 °C for metabolomics analysis. After homogenizing with UltraTurrax (IKA T25, IKA-Werke GmbH & Co. KG, Staufen, Germany), 250 mg of each treatment’s samples were extracted in acidified 80% methanol for metabolites suspension, centrifuged, and filtered through a 0.22 μm cellulose membrane into vials for analysis. Untargeted metabolomic analysis and metabolite identification in sample extracts were carried out at oloBion Laboratory (Barcelona, Spain) using an ultra-high performance liquid chromatograph (UHPLC) coupled to a quadrupole-time-of-flight mass spectrometer (UHPLC/QTOF-MS) (Agilent Technologies, Santa Clara, CA, USA) according to the procedure described by Bonini et al. [62]. The detected compounds were identified and classified using the PlantCyc 16.0.2 database (Plant Metabolic Network, Michigan State University).

### 4.10. Statistical Analysis

A two-way analysis of variance (ANOVA) and Tukey’s post hoc test were per-formed with R packages (RStudio Team ‘Cranberry Hibiscus’, Wien, Austria) to evaluate the significance of the main effects of the two irrigation levels (well-watered and water-stressed), the biostimulant treatments (untreated control, PH applied foliarly, and PH applied via root drench) and their interaction. When significant effects were detected, Tukey’s HSD test (*p* = 0.05) was applied to compare means and identify statistically distinct treatment groups.

Metabolic data was processed using oloMAP 2.02 developed at the oloBion Com-pany [62].

## 5. Conclusions

Well-watering enhanced biomass accumulation and fruit yield as compared to stressed plants. However, water stress improved certain fruit quality traits, such as sugar content, antioxidant capacity, and firmness. While previous studies with vegetal-derived protein hydrolysates (PH) proved enhanced plant resilience to drought, their application in the current experiment did not produce significant improvements in biomass or fruit yield, possibly due to the severity of drought conditions that exceeded the threshold for effective biostimulant action. The PH application method (foliar or root drench) had only transient effects on physiological responses, without alleviating the effects of prolonged drought. Metabolomics analysis unravelled the increase in key metabolites involved in stress response and fruit quality, such as amino acids, antioxidants, and carotenoid derivatives.

However, PH treatments—regardless of application method—did not result in significant changes in fruit metabolic composition. These findings underscore the importance of carefully considering the timing of PH application and the extent of drought stress. In fact, the observed potential of water stress to enhance specific quality traits in tomato fruits highlight the need for future research to refine PH application strategies under variable water stress conditions.

## Figures and Tables

**Table 1:** Yield traits (marketable, not-marketable fruits and total yield; number and weight of marketable fruits) of tomato plants in response to irrigation and biostimulant treatments. W = watered; S = stressed; U = untreated; PH-F = foliar; PH-R = root. ns, ***, non-significant or significant at *p* ≤ 0.001. Values are presented as mean ± standard error.

Source of Variation	Yield (g/Plant)			Marketable Fruits	
	Marketable	Not Marketable	Total	Number (n./Plant)	Mean Weight (g/Fruit)
***Irrigation***					
W	2484.32 ± 40.49	528.60 ± 22.26	3012.35 ± 51.37	152.98 ± 2.65	16.41 ± 0.16
S	1471.79 ± 23.49	368.83 ± 11.18	1798.64 ± 23.32	103.98 ± 1.49	14.39 ± 0.13
***Biostimulant***					
U	2078.02 ± 121.57	434.48 ± 19.60	2454.01 ± 124.56	131.83 ± 6.00	15.41 ± 0.24
PH-F	1971.25 ± 94.03	462.10 ± 27.88	2345.24 ± 106.5	129.41 ± 4.57	15.25 ± 0.23
PH-R	1915.33 ± 97.02	447.02 ± 29.24	2436.41 ± 133.11	125.06 ± 4.95	15.55 ± 0.30
***Irrigation*** × ***Biostimulant***					
W × U	2614.52 ± 66.40	471.52 ± 29.99	3031.71 ± 86.46	157.94 ± 5.40	16.49 ± 0.20
W × PH-F	2420.29 ± 71.90	567.67 ± 38.49	2932.55 ± 49.57	150.75 ± 4.44	16.09 ± 0.28
W × PH-R	2427.31 ± 62.47	547.81 ± 44.61	3062.80 ± 113.39	150.07 ± 3.66	16.64 ± 0.33
S × U	1452.11 ± 23.46	397.44 ± 22.47	1793.79 ± 37.98	102.00 ± 2.34	14.40 ± 0.20
S × PH-F	1492.28 ± 38.69	356.53 ± 15.70	1831.35 ± 39.04	108.06 ± 2.39	14.30 ± 0.11
S × PH-R	1467.34 ± 49.93	352.54 ± 18.48	1768.27 ± 44.58	101.63 ± 2.76	14.45 ± 0.33
***Significance***					
Irrigation	***	***	***	***	***
Biostimulant	ns	ns	ns	ns	ns
Irrigation × Biostimulant	ns	ns	ns	ns	ns

**Table 2:** Leaf pigment concentration for chlorophyll a, chlorophyll b, total chlorophyll, and carotenoid content in tomato leaves sampled at mid-crop cycle. DAT = days after transplanting; W = watered; S = stressed; U = untreated; PH-F = foliar; PH-R = root. ns, *, ***, non-significant or significant at *p* ≤ 0.05 or 0.001. Different letters indicate significantly different means (Tukey’s HSD test for *p* = 0.05). Values are presented as mean ± standard error.

Source of Variation	Pigment Concentration (mg/g FW)			
	Clorophyll a	Clorophyll b	Total Chlorophyll	Carotenoids
***Irrigation***				
W	1.188 ± 0.013	0.450 ± 0.004	1.637 ± 0.017	0.333 ± 0.004
S	1.359 ± 0.021	0.500 ± 0.005	1.858 ± 0.027	0.365 ± 0.005
***Biostimulant***				
U	1.285 ± 0.022 ab	0.479 ± 0.006 ab	1.764 ± 0.027 ab	0.351 ± 0.005
PH-F	1.227 ± 0.017 b	0.462 ± 0.005 b	1.688 ± 0.022 b	0.341 ± 0.005
PH-R	1.305 ± 0.030 a	0.483 ± 0.008 a	1.788 ± 0.039 a	0.355 ± 0.007
***Irrigation*** × ***Biostimulant***				
W × U	1.232 ± 0.023 bc	0.463 ± 0.007	1.695 ± 0.030 bc	0.342 ± 0.006
W × PH-F	1.151 ± 0.019 c	0.438 ± 0.005	1.589 ± 0.023 c	0.325 ± 0.006
W × PH-R	1.181 ± 0.023 c	0.447 ± 0.007	1.628 ± 0.030 c	0.332 ± 0.006
S × U	1.337 ± 0.034 ab	0.494 ± 0.009	1.831 ± 0.043 ab	0.359 ± 0.007
S × PH-F	1.306 ± 0.022 b	0.486 ± 0.006	1.790 ± 0.028 b	0.357 ± 0.006
S × PH-R	1.430 ± 0.046 a	0.518 ± 0.013	1.948 ± 0.059 a	0.378 ± 0.010
**Significance**				
Irrigation	***	***	***	***
Biostimulant	*	*	*	ns
Irrigation × Biostimulant	*	ns	*	ns

**Table 3:** Quality parameters (Total soluble solids—TSS, pH, titratable acidity—TA, dry matter—DM, and firmness) of marketable fruits measured at the IV truss in response to irrigation (watered vs. stressed) and biostimulant (untreated, foliar, root) treatments. W = watered; S = stressed; U = untreated; PH-F = foliar; PH-R = root. ns, *, ***, non-significant or significant at *p* ≤ 0.05, or 0.001. Values are presented as mean ± standard error.

Source of Variation	pH	TSS (°Brix)	TA (g Citric Acid/100 mL Juice)	TSS/TA	DM (%)	Firmness (N)
***Irrigation***						
W	4.32 ± 0.01	8.55 ± 0.05	0.46 ± 0.01	18.81 ± 0.24	9.30 ± 0.08	0.95 ± 0.02
S	4.29 ± 0.01	8.83 ± 0.04	0.48 ± 0.01	18.65 ± 0.29	9.72 ± 0.07	1.09 ± 0.02
***Biostimulant***						
U	4.31 ± 0.01	8.65 ± 0.06	0.46 ± 0.01	18.99 ± 0.35	9.49 ± 0.08	1.02 ± 0.02
PH-F	4.30 ± 0.01	8.76 ± 0.06	0.48 ± 0.01	18.44 ± 0.25	9.54 ± 0.10	1.00 ± 0.03
PH-R	4.32 ± 0.01	8.66 ± 0.07	0.47 ± 0.01	18.74 ± 0.35	9.38 ± 0.12	1.02 ± 0.03
***Irrigation* × *Biostimulant***						
W × U	4.34 ± 0.02	8.49 ± 0.11	0.46 ± 0.01	18.90 ± 0.51	9.37 ± 0.10	0.99 ± 0.04
W × PH-F	4.31 ± 0.02	8.58 ± 0.07	0.45 ± 0.01	19.11 ± 0.26	9.39 ± 0.14	0.91 ± 0.04
W × PH-R	4.33 ± 0.01	8.58 ± 0.09	0.47 ± 0.01	18.47 ± 0.43	9.14 ± 0.16	0.95 ± 0.02
S × U	4.27 ± 0.02	8.79 ± 0.05	0.47 ± 0.01	19.08 ± 0.48	9.64 ± 0.13	1.05 ± 0.02
S × PH-F	4.29 ± 0.02	8.94 ± 0.08	0.50 ± 0.01	17.82 ± 0.37	9.78 ± 0.07	1.12 ± 0.02
S × PH-R	4.30 ± 0.02	8.77 ± 0.09	0.47 ± 0.01	19.06 ± 0.58	9.76 ± 0.13	1.09 ± 0.04
***Significance***						
Irrigation	*	***	*	ns	***	***
Biostimulant	ns	ns	ns	ns	ns	ns
Irrigation × Biostimulant	ns	ns	ns	ns	ns	ns

**Table 4:** Concentrations of macronutrients (N, P, K) and micronutrients (Fe, Mn, Zn, Cu) in tomato fruits in response to irrigation (watered vs. stressed) and biostimulant (untreated, foliar, root) treatments. W = watered; S = stressed; U = untreated; PH-F = foliar; PH-R = root. ns, *, ***, non-significant or significant at *p* ≤ 0.05 or 0.001. Different letters indicate significantly different means (Tukey’s HSD test for *p* = 0.05). Values are presented as mean ± standard error.

Source of Variation	Macronutrient Concentration (g/kg DW)			Micronutrient Concentration (mg/kg DW)			
	N	P	K	Fe	Mn	Zn	Cu
***Irrigation***							
W	21.85 ± 0.32	7.86 ± 0.43	50.64 ± 0.86	304.53 ± 10.53	62.74 ± 2.61	25.44 ± 0.83	5.63 ± 0.15
S	21.62 ± 0.30	6.85 ± 0.63	40.75 ± 1.26	280.66 ± 18.75	42.81 ± 1.45	18.17 ± 0.51	5.07 ± 0.16
***Biostimulant***							
U	21.49 ± 0.36	8.03 ± 0.64	43.93 ± 1.90	250.01 ± 14.36 b	52.77 ± 3.84	21.93 ± 1.61	5.52 ± 0.24
PH-F	21.86 ± 0.44	7.21 ± 0.76	47.59 ± 2.17	325.85 ± 13.38 a	57.71 ± 4.82	22.21 ± 1.49	5.23 ± 0.27
PH-R	21.85 ± 0.34	6.68 ± 0.65	44.98 ± 1.97	303.05 ± 19.52 ab	51.57 ± 3.84	21.63 ± 1.17	5.25 ± 0.11
***Irrigation* × *Biostimulant***							
W × U	21.82 ± 0.59	8.15 ± 0.76	49.55 ± 1.38	266.78 ± 14.98	61.95 ± 4.38	25.15 ± 2.21	6.02 ± 0.13
W × PH-F	21.48 ± 0.63	7.83 ± 0.88	52.90 ± 0.48	314.57 ± 16.91	64.59 ± 5.20	25.99 ± 1.02	5.52 ± 0.36
W × PH-R	22.14 ± 0.56	7.48 ± 0.49	49.67 ± 1.81	325.95 ± 14.82	61.69 ± 4.73	25.19 ± 1.03	5.46 ± 0.09
S × U	21.15 ± 0.41	7.90 ± 1.09	39.25 ± 1.51	236.03 ± 22.81	43.60 ± 3.49	18.07 ± 0.26	5.19 ± 0.33
S × PH-F	22.15 ± 0.67	6.47 ± 1.32	42.28 ± 2.61	342.77 ± 21.66	43.95 ± 1.21	17.67 ± 1.09	4.90 ± 0.38
S × PH-R	21.57 ± 0.42	6.22 ± 0.96	40.95 ± 2.48	283.41 ± 33.44	41.46 ± 1.23	18.59 ± 1.00	5.10 ± 0.17
***Significance***							
Irrigation	ns	ns	***	ns	***	***	*
Biostimulant	ns	ns	ns	*	ns	ns	ns
Irrigation × Biostimulant	ns	ns	ns	ns	ns	ns	ns

**Table 5:** Antioxidant capacity of tomato fruit tissues expressed as Ferric Reducing Antioxidant Power (FRAP), 2,2-diphenyl-1-picrylhydrazyl (DPPH) in mg Trolox equivalents/g fruit fresh weight—FW, total polyphenols as mg gallic acid equivalents—GAE/g FW, and flavonoids as mg quercetin equivalents—QE/g FW. W = watered; S = stressed; U = untreated; PH-F = foliar; PH-R = root. ns, *, ***, non-significant or significant at *p* ≤ 0.05 or 0.001. Different letters indicate significantly different means (Tukey’s HSD test for *p* = 0.05). Values are presented as mean ± standard error.

Source of Variation	mg Trolox eq./g FW		mg GAE eq./g FW	mg QE eq./g FW
	FRAP	DPPH	Polyphenols	Flavonoids
***Irrigation***				
W	22.106 ± 0.593	11.742 ± 0.077	31.576 ± 0.646	0.252 ± 0.016
S	25.935 ± 0.437	11.519 ± 0.109	33.817 ± 0.657	0.304 ± 0.016
***Biostimulant***				
U	22.293 ± 0.582 b	11.657 ± 0.128	32.141 ± 0.921	0.254 ± 0.021
PH-F	26.034 ± 0.891 a	11.699 ± 0.136	32.844 ± 0.785	0.278 ± 0.025
PH-R	23.706 ± 0.496 b	11.516 ± 0.072	33.104 ± 0.897	0.311 ± 0.011
***Irrigation*** × ***Biostimulant***				
W × U	19.622 ± 0.414	11.797 ± 0.116	30.864 ± 1.184	0.247 ± 0.039
W × PH-F	23.779 ± 1.304	11.753 ± 0.173	31.701 ± 1.014	0.243 ± 0.026
W × PH-R	22.581 ± 0.690	11.662 ± 0.106	32.162 ± 1.294	0.271 ± 0.008
S × U	24.519 ± 0.406	11.518 ± 0.228	33.418 ± 1.297	0.261 ± 0.020
S × PH-F	28.740 ± 0.618	11.645 ± 0.216	33.987 ± 1.078	0.313 ± 0.040
S × PH-R	24.831 ± 0.574	11.371 ± 0.082	34.047 ± 1.229	0.338 ± 0.006
***Significance***				
Irrigation	***	ns	*	*
Biostimulant	***	ns	ns	ns
Irrigation × Biostimulant	ns	ns	ns	ns

**Table 6:** Concentration of glucose, sucrose, fructose, starch, and total soluble carbohydrates (mg/g DW) in tomato fruits in response to irrigation (watered vs. stressed) and biostimulant (untreated, foliar, root) treatments. W = watered; S = stressed; U = untreated; PH-F = foliar; PH-R = root. ns, *, **, non-significant or significant at *p* ≤ 0.05, or 0.01. Different letters indicate significantly different means (Tukey’s HSD test for *p* = 0.05). Values are presented as mean ± standard error.

Source of Variation	Soluble Carbohydrates (mg/g DW)				Insoluble Carbohydrate (mg/g DW)
	Glucose	Sucrose	Fructose	Total	Starch
***Irrigation***					
W	233.62 ± 12.82	27.22 ± 2.30	296.91 ± 22.13	557.76 ± 34.13	6.03 ± 1.40
S	271.47 ± 7.44	20.20 ± 2.50	325.04 ± 16.22	616.71 ± 23.37	4.88 ± 0.58
***Biostimulant***					
U	266.25 ± 10.88	18.48 ± 3.68	312.66 ± 24.18	597.34 ± 35.04	4.18 ± 0.72
PH-F	233.52 ± 12.63	25.50 ± 2.20	298.60 ± 17.56	557.62 ± 26.98	6.26 ± 2.10
PH-R	257.87 ± 18.68	27.16 ± 2.86	321.67 ± 31.28	606.69 ± 49.00	5.92 ± 0.50
***Irrigation*** × ***Biostimulant***					
W × U	272.94 ± 20.66 ab	23.74 ± 3.52	331.04 ± 50.30	627.71 ± 71.98	3.42 ± 0.07
W × PH-F	208.40 ± 12.81 c	27.98 ± 3.58	291.28 ± 34.69	527.65 ± 49.70	8.67 ± 3.99
W × PH-R	219.53 ± 13.80 bc	29.97 ± 5.32	268.41 ± 33.74	517.91 ± 51.34	5.10 ± 0.79
S × U	259.57 ± 10.95 abc	13.22 ± 5.27	294.29 ± 7.54	567.08 ± 6.31	4.95 ± 1.42
S × PH-F	258.65 ± 1.48 abc	23.03 ± 2.29	305.92 ± 16.87	587.59 ± 16.49	3.85 ± 0.61
S × PH-R	296.21 ± 9.14 a	24.34 ± 2.19	374.92 ± 30.28	695.47 ± 38.54	5.83 ± 0.77
***Significance***					
Irrigation	**	*	ns	ns	ns
Biostimulant	ns	ns	ns	ns	ns
Irrigation × Biostimulant	*	ns	ns	ns	ns

## Data Availability

Data will be made available on request.

## Supplementary Materials

The following supporting information can be downloaded at: https://www.mdpi.com/article/10.3390/plants14132000/s1, Supplementary Table S1: Dry weight (DW) of leaves, stems, fruits, and above-ground biomass of tomato plants in response to irrigation and biostimulant treatments. Above-ground biomass is the sum of leaves, stems, and fruit DW. W = watered; S = stressed; U = untreated; PH-F = foliar; PH-R = root. ns, ***, non-significant or significant at *p* ≤ 0.001. Values are presented as mean ± standard error. Supplementary Table S2: Non-destructive measurements of chlorophyll index with MPM100 on tomato leaves in response to irrigation and biostimulant treatments. DAT = days after transplanting; W = watered; S = stressed; U = untreated; PH-F = foliar; PH-R = root. ns, *, ***, non-significant or significant at *p* ≤ 0.05 or 0.001. Values are presented as mean ± standard error. Supplementary Table S3: Diameter measured on marketable fruits for all stages (I-VIII truss). W = watered; S = stressed; U = untreated; PH-F = foliar; PH-R = root. ns, **, ***, non-significant or significant at *p* ≤ 0.01 or 0.001. Values are presented as mean ± standard error.

## References

[1] Furtak K., Wolińska A. (2023). The Impact of Extreme Weather Events as a Consequence of Climate Change on the Soil Moisture and on the Quality of the Soil Environment and Agriculture—A Review. Catena.

[2] Nikolaou G., Neocleous D., Christou A., Kitta E., Katsoulas N. (2020). Implementing Sustainable Irrigation in Water-Scarce Regions under the Impact of Climate Change. Agronomy.

[3] Du Y., Zhao Q., Chen L., Yao X., Zhang W., Zhang B., Xie F. (2020). Effect of Drought Stress on Sugar Metabolism in Leaves and Roots of Soybean Seedlings. Plant Physiol. Biochem..

[4] Wahab A., Abdi G., Saleem M.H., Ali B., Ullah S., Shah W., Mumtaz S., Yasin G., Muresan C.C., Marc R.A. (2022). Alleviate the Adverse Effects of Drought Stress: A Comprehensive Review. Plants.

[5] Razi K., Muneer S. (2021). Drought Stress-Induced Physiological Mechanisms, Signaling Pathways and Molecular Response of Chloroplasts in Common Vegetable Crops. Crit. Rev. Biotechnol..

[6] Apel K., Hirt H. (2004). Reactive Oxygen Species: Metabolism, Oxidative Stress, and Signal Transduction. Annu. Rev. Plant Biol..

[7] Triantaphylidès C., Havaux M. (2009). Singlet Oxygen in Plants: Production, Detoxification and Signaling. Trends Plant Sci..

[8] Beveridge T., Loubert E., Harrison J.E. (2000). Simple Measurement of Phenolic Esters in Plant Cell Walls. Food Res. Int..

[9] Solimene S., Coluccia D., Bernardo A. (2023). Environmental Impact of Different Business Models: An LCA Study of Fresh Tomato Production in Italy. Sustainability.

[10] Kalra A., Goel S., Elias A.A. (2024). Understanding Role of Roots in Plant Response to Drought: Way Forward to Climate-Resilient Crops. Plant Genome.

[11] Hou X., Zhang W., Du T., Kang S., Davies W.J. (2020). Responses of Water Accumulation and Solute Metabolism in Tomato Fruit to Water Scarcity and Implications for Main Fruit Quality Variables. J. Exp. Bot..

[12] Barbagallo R.N., Di Silvestro I., Patanè C. (2013). Yield, Physicochemical Traits, Antioxidant Pattern, Polyphenol Oxidase Activity and Total Visual Quality of Field-Grown Processing Tomato Cv. Brigade as Affected by Water Stress in Mediterranean Climate. J. Sci. Food Agric..

[13] Jin N., Jin L., Wang S., Meng X., Ma X., He X., Zhang G., Luo S., Lyu J., Yu J. (2022). A Comprehensive Evaluation of Effects on Water-Level Deficits on Tomato Polyphenol Composition, Nutritional Quality and Antioxidant Capacity. Antioxidants.

[14] Colla G., Rouphael Y., Canaguier R., Svecova E., Cardarelli M. (2014). Biostimulant Action of a Plant-Derived Protein Hydrolysate Produced through Enzymatic Hydrolysis. Front. Plant Sci..

[15] Francesca S., Cirillo V., Raimondi G., Maggio A., Barone A., Rigano M.M. (2021). A Novel Protein Hydrolysate-Based Biostimulant Improves Tomato Performances under Drought Stress. Plants.

[16] Agliassa C., Mannino G., Molino D., Cavalletto S., Contartese V., Margherita C., Secchi F. (2021). A New Protein Hydrolysate-Based Biostimulant Applied by Fertigation Promotes Relief from Drought Stress in Capsicum Annuum L.. Plant Physiol. Biochem..

[17] Leporino M., Rouphael Y., Bonini P., Colla G., Cardarelli M. (2024). Protein Hydrolysates Enhance Recovery from Drought Stress in Tomato Plants: Phenomic and Metabolomic Insights. Front. Plant Sci..

[18] Paul K., Sorrentino M., Lucini L., Rouphael Y., Cardarelli M., Bonini P., Miras Moreno M.B., Reynaud H., Canaguier R., Trtílek M. (2019). A Combined Phenotypic and Metabolomic Approach for Elucidating the Biostimulant Action of a Plant-Derived Protein Hydrolysate on Tomato Grown under Limited Water Availability. Front. Plant Sci..

[19] Patanè C., Tringali S., Sortino O. (2011). Effects of Deficit Irrigation on Biomass, Yield, Water Productivity and Fruit Quality of Processing Tomato under Semi-Arid Mediterranean Climate Conditions. Sci. Hortic..

[20] Ahmed M., Ullah H., Piromsri K., Tisarum R., Cha-um S., Datta A. (2022). Effects of an *Ascophyllum nodosum* Seaweed Extract Application Dose and Method on Growth, Fruit Yield, Quality, and Water Productivity of Tomato under Water-Deficit Stress. S. Afr. J. Bot..

[21] Zhao W., Liu L., Shen Q., Yang J., Han X., Tian F., Wu J. (2020). Effects of Water Stress on Photosynthesis, Yield, and Water Use Efficiency in Winter Wheat. Water.

[22] Rosa A.P., Barão L., Chambel L., Cruz C., Santana M.M. (2023). Early Identification of Plant Drought Stress Responses: Changes in Leaf Reflectance and Plant Growth Promoting Rhizobacteria Selection-The Case Study of Tomato Plants. Agronomy.

[23] Makela P., Karkkainen J., Somersalo S. (2000). Effect of Glycinebetaine on Chloroplast Ultrastructure, Chlorophyll and Protein Content, and RuBPCO Activities in Tomato Grown under Drought or Salinity. Biol. Plant..

[24] Rodriguez J., Rios D., Rodriguez E., Diaz C. (2006). Physico-Chemical Changes During Ripening of Conventionally, Ecologically and Hydroponically Cultivated Tyrlain (TY 10016) Tomatoes. Int. J. Agric. Res..

[25] Ripoll J., Urban L., Brunel B., Bertin N. (2016). Water Deficit Effects on Tomato Quality Depend on Fruit Developmental Stage and Genotype. J. Plant Physiol..

[26] Mitchell J.P., Shennan C., Grattan S.R., May D.M. (2019). Tomato Fruit Yields and Quality under Water Deficit and Salinity. J. Am. Soc. Hortic. Sci..

[27] Levander O.A. (1990). Fruit and Vegetable Contributions to Dietary Mineral Intake in Human Health and Disease. HortScience.

[28] Mitchell J.P., Shennan C., Grattan S.R. (1991). Developmental Changes in Tomato Fruit Composition in Response to Water Deficit and Salinity. Physiol. Plant..

[29] Ahmed N., Zhang B., Chachar Z., Li J., Xiao G., Wang Q., Hayat F., Deng L., Narejo M.N., Bozdar B. (2024). Micronutrients and Their Effects on Horticultural Crop Quality, Productivity and Sustainability. Sci. Hortic..

[30] Rai S., Singh P.K., Mankotia S., Swain J., Satbhai S.B. (2021). Iron Homeostasis in Plants and Its Crosstalk with Copper, Zinc, and Manganese. Plant Stress..

[31] Lu S.W., Li T.L., Jiang J. (2009). Tomato Key Sucrose Metabolizing Enzyme Activities and Gene Expression Under NaCl and PEG Iso-Osmotic Stresses. Agric. Sci. China.

[32] Luo A., Zhou C., Chen J. (2021). The Associated With Carbon Conversion Rate and Source–Sink Enzyme Activity in Tomato Fruit Subjected to Water Stress and Potassium Application. Front. Plant Sci..

[33] Singh P., Choudhary K.K., Chaudhary N., Gupta S., Sahu M., Tejaswini B., Sarkar S. (2022). Salt Stress Resilience in Plants Mediated through Osmolyte Accumulation and Its Crosstalk Mechanism with Phytohormones. Front. Plant Sci..

[34] Rai V.K. (2002). Role of Amino Acids in Plant Responses to Stress. Biol. Plant..

[35] Kishor P.B.K., Hima Kumari P., Sunita M.S.L., Sreenivasulu N. (2015). Role of Proline in Cell Wall Synthesis and Plant Development and Its Implications in Plant Ontogeny. Front. Plant Sci..

[36] Galili G., Tang G., Zhu X., Gakiere B. (2001). Lysine Catabolism: A Stress and Development Super-Regulated Metabolic Pathway. Curr. Opin. Plant Biol..

[37] Zhang X., Ji N., Zhen F., Ren P., Li F. (2014). Metabolism of Endogenous Arginine in Tomato Fruit Harvested at Different Ripening Stages. Sci. Hortic..

[38] Gao F., Mei X., Li Y., Guo J., Shen Y. (2021). Update on the Roles of Polyamines in Fleshy Fruit Ripening, Senescence, and Quality. Front. Plant Sci..

[39] Dabravolski S.A., Isayenkov S.V. (2023). The Role of Anthocyanins in Plant Tolerance to Drought and Salt Stresses. Plants.

[40] Hou X., Rivers J., León P., McQuinn R.P., Pogson B.J. (2016). Synthesis and Function of Apocarotenoid Signals in Plants. Trends Plant Sci..

[41] Simkin A.J. (2021). Carotenoids and Apocarotenoids in Planta: Their Role in Plant Development, Contribution to the Flavour and Aroma of Fruits and Flowers, and Their Nutraceutical Benefits. Plants.

[42] Gosch C., Halbwirth H., Stich K. (2010). Phloridzin: Biosynthesis, Distribution and Physiological Relevance in Plants. Phytochemistry.

[43] Schijlen E., Ric De Vos C.H., Jonker H., Van Den Broeck H., Molthoff J., Van Tunen A., Martens S., Bovy A. (2006). Pathway Engineering for Healthy Phytochemicals Leading to the Production of Novel Flavonoids in Tomato Fruit. Plant Biotechnol. J..

[44] Tohge T., Scossa F., Wendenburg R., Frasse P., Balbo I., Watanabe M., Alseekh S., Jadhav S.S., Delfin J.C., Lohse M. (2020). Exploiting Natural Variation in Tomato to Define Pathway Structure and Metabolic Regulation of Fruit Polyphenolics in the Lycopersicum Complex. Mol. Plant.

[45] Dere S., Kusvuran S., Dasgan H.Y. (2022). Does Drought Increase the Antioxidant Nutrient Capacity of Tomatoes?. Int. J. Food Sci. Technol..

[46] Hano S., Shibuya T., Imoto N., Ito A., Imanishi S., Aso H., Kanayama Y. (2017). Scientia Horticulturae Serotonin Content in Fresh and Processed Tomatoes and Its Accumulation during Fruit Development. Sci. Hortic..

[47] Conti V., Romi M., Guarnieri M., Cantini C., Cai G. (2022). Italian Tomato Cultivars under Drought Stress Show Different Content of Bioactives in Pulp and Peel of Fruits. Foods.

[48] Tieman D., Taylor M., Schauer N., Fernie A.R., Hanson A.D., Klee H.J. (2006). Tomato Aromatic Amino Acid Decarboxylases Participate in Synthesis of the Flavor Volatiles 2-Phenylethanol and 2-Phenylacetaldehyde. Proc. Natl. Acad. Sci. USA.

[49] Shohat H., Eliaz N.I., Weiss D. (2021). Gibberellin in Tomato: Metabolism, Signaling and Role in Drought Responses. Mol. Hortic..

[50] Park M., Malka S.K. (2022). Gibberellin Delays Metabolic Shift during Tomato Ripening by Inducing Auxin Signaling. Front. Plant Sci..

[51] Nir I., Moshelion M., Weiss D., Transferase M. (2014). The Arabidopsis GIBBERELLIN METHYL TRANSFERASE 1 Suppresses Gibberellin Activity, Reduces Whole-Plant Transpiration and Promotes Drought Tolerance in Transgenic Tomato. Plant Cell Environ..

[52] Zhao W., Huang H., Jingjing W., Xiaoyun W., Bingqin X., Xuehui Y., Lulu S., Rui Y., Jianli W., Aidong S. (2023). Jasmonic Acid Enhances Osmotic Stress Responses by MYC2- Mediated Inhibition of Protein Phosphatase 2C1 and Response Regulators 26 Transcription Factor in Tomato. Plant J..

[53] El-Nakhel C., Cristofano F., Colla G., Pii Y., Secomandi E., De Gregorio M., Buffagni V., Garcia-Perez P., Lucini L., Rouphael Y. (2023). Vegetal-Derived Biostimulants Distinctively Command the Physiological and Metabolomic Signatures of Lettuce Grown in Depleted Nitrogen Conditions. Sci. Hortic..

[54] Wellburn A.R. (1994). The Spectral Determination of Chlorophylls a and b, as Well as Total Carotenoids, Using Various Solvents with Spectrophotometers of Different Resolution. J. Plant Physiol..

[55] Stadtman F.H., Buhlert J.E., Marsh G.L. (1977). Titratable Acidity of Tomato Juice As Affected By Break Procedure. J. Food Sci..

[56] Bremner J.M. (1965). Total Nitrogen. Am. Soc. Agron..

[57] Benzie I.F.F., Strain J.J. (1967). The Ferric Reducing Ability of Plasma (FRAP) as a Measure of “Antioxidant Power”: The FRAP Assay. Verh. Dtsch. Ges. Inn. Med..

[58] Brand-Williams W., Cuvelier M.E., Berset C. (1995). Use of a Free Radical Method to Evaluate Antioxidant Activity. LWT Food Sci. Technol..

[59] Lombardo S., Pandino G., Ierna A., Mauromicale G. (2012). Variation of Polyphenols in a Germplasm Collection of Globe Artichoke. Food Res. Int..

[60] Zhishen J., Mengcheng T., Jianming W. (1999). The Determination of Flavonoid Contents in Mulberry and Their Scavenging Effects on Superoxide Radicals. Food Chem..

[61] Adsule P., Dan A. (1979). Simplified Extraction Procedure in the Rapid Spectrophotometric Method for Lycopene Estimation in Tomato. J. Food Sci. Technol..

[62] Bonini P., Kind T., Tsugawa H., Barupal D.K., Fiehn O. (2020). Retip: Retention Time Prediction for Compound Annotation in Untargeted Metabolomics. Anal. Chem..

