# Supplementary material for: Impact of Drought and Biostimulant in Greenhouse Tomato: Agronomic and Metabolomic Insights
Source: Plants (Basel). 2025 Jun 30;14(13):2000. doi: 10.3390/plants14132000 (PMC12251554; doi:10.3390/plants14132000)
Supplement: Supplementary file 1 [file plants-14-02000-s001.zip › plants-3693342-supplementary.pdf]

Supplementary Table 1

Source of variation	Dry weight (g/plant)			
	Leaves	Stem	Fruits	Above-ground biomass
Irrigation				
Watered (W)	149.86 ± 2.23	82.73 ± 1.11	249.05 ± 6.60	484.28 ± 7.85
Stressed (S)	96.01 ± 1.78	55.24 ± 1.00	157.35 ± 3.65	308.13 ± 5.09
Biostimulant				
Untreated (U)	121.20 ± 5.59	67.82 ± 2.88	204.48 ± 10.86	402.30 ± 18.04
Foliar (PH-F)	125.26 ± 5.51	70.04 ± 2.57	203.96 ± 8.91	397.27 ± 15.63
Root (PH-R)	124.06 ± 5.63	68.62 ± 2.98	204.10 ± 11.87	394.92 ± 19.96
IrrigationxBiostimulant				
WxU	148.26 ± 3.43	81.66 ± 1.95	243.63 ± 14.36	478.10 ± 17.48
WxPH-F	149.95 ± 2.84	82.85 ± 1.97	240.92 ± 9.61	476.56 ± 10.60
WxPH-R	151.29 ± 5.01	83.61 ± 1.96	262.61 ± 9.59	498.18 ± 11.94
SxU	94.14 ± 3.59	53.99 ± 1.82	162.71 ± 6.76	315.68 ± 8.44
SxPH-F	94.19 ± 1.96	58.02 ± 1.55	164.55 ± 5.61	317.98 ± 7.69
SxPH-R	99.23 ± 3.16	53.63 ± 1.73	145.58 ± 5.78	291.66 ± 9.07
Significance				
Irrigation	***	***	***	***
Biostimulant	ns	ns	ns	ns
IrrigationxBiostimulant	ns	ns	ns	ns

Supplementary Table 2

Source of variation	Chlorophyll index				
	20 DAT	55 DAT	84 DAT	112 DAT	133 DAT
Irrigation					
W	0.428 ± 0.004	0.837 ± 0.009	0.714 ± 0.008	0.869 ± 0.009	1.040 ± 0.01
S	0.421 ± 0.004	0.886 ± 0.007	0.894 ± 0.011	0.924 ± 0.008	1.175 ± 0.014
Biostimulant					
U	0.418 ± 0.005	0.883 ± 0.011 a	0.806 ± 0.014	0.907 ± 0.009	1.099 ± 0.015 b
PH-F	0.429 ± 0.005	0.853 ± 0.009 ab	0.799 ± 0.014	0.901 ± 0.011	1.157 ± 0.017 a
PH-R	0.427 ± 0.005	0.847 ± 0.011 b	0.806 ± 0.017	0.882 ± 0.011	1.063 ± 0.015 b
IrrigationxBiostimulant					
WxU	0.413 ± 0.008 ab	0.861 ± 0.018 ab	0.730 ± 0.015	0.892 ± 0.014	1.057 ± 0.019 cd
WxPH-F	0.430 ± 0.007 ab	0.857 ± 0.014 ab	0.715 ± 0.014	0.858 ± 0.015	1.060 ± 0.018 bcd
WxPH-R	0.442 ± 0.006 a	0.793 ± 0.013 c	0.697 ± 0.015	0.857 ± 0.018	1.000 ± 0.014 d
SxU	0.423 ± 0.007 ab	0.906 ± 0.011 a	0.878 ± 0.018	0.922 ± 0.012	1.140 ± 0.022 b
SxPH-F	0.429 ± 0.007 ab	0.849 ± 0.011 b	0.887 ± 0.017	0.944 ± 0.013	1.253 ± 0.021 a
SxPH-R	0.412 ± 0.008 b	0.904 ± 0.012 ab	0.916 ± 0.022	0.906 ± 0.014	1.129 ± 0.023 bc
Significance					
Irrigation	ns	*	***	***	***
Biostimulant	ns	***	ns	ns	***
IrrigationxBiostimulant	*	***	ns	ns	*

Supplementary Table 3

Source of variation	Fruit diameter (cm)							
	I	II	III	IV	V	VI	VII	VIII
Irrigation								
W	3.62 ± 0.02	3.60 ± 0.01	3.54 ± 0.02	3.19 ± 0.02	3.12 ± 0.01	3.09 ± 0.01	3.17 ± 0.01	3.06 ± 0.01
S	3.39 ± 0.02	3.29 ± 0.02	3.27 ± 0.02	3.10 ± 0.01	3.00 ± 0.01	2.99 ± 0.01	2.98 ± 0.01	2.99 ± 0.01
Biostimulant								
U	3.52 ± 0.03	3.47 ± 0.03	3.41 ± 0.03	3.13 ± 0.02 b	3.07 ± 0.02	3.05 ± 0.01	3.08 ± 0.02	3.03 ± 0.01
PH-F	3.50 ± 0.03	3.40 ± 0.03	3.38 ± 0.03	3.11 ± 0.02 b	3.05 ± 0.02	3.03 ± 0.02	3.07 ± 0.02	3.03 ± 0.01
PH-R	3.52 ± 0.03	3.45 ± 0.04	3.41 ± 0.04	3.20 ± 0.02 a	3.07 ± 0.02	3.05 ± 0.02	3.08 ± 0.03	3.02 ± 0.02
IrrigationxBiostimulant								
WxU	3.65 ± 0.03	3.62 ± 0.02	3.56 ± 0.01	3.17 ± 0.03	3.14 ± 0.02	3.09 ± 0.02	3.16 ± 0.02	3.05 ± 0.02
WxPH-F	3.59 ± 0.03	3.55 ± 0.03	3.49 ± 0.04	3.15 ± 0.02	3.08 ± 0.02	3.06 ± 0.03	3.14 ± 0.03	3.06 ± 0.02
WxPH-R	3.62 ± 0.04	3.62 ± 0.02	3.56 ± 0.02	3.26 ± 0.03	3.13 ± 0.03	3.11 ± 0.02	3.20 ± 0.02	3.08 ± 0.03
SxU	3.36 ± 0.02	3.32 ± 0.04	3.27 ± 0.02	3.09 ± 0.03	2.98 ± 0.01	3.00 ± 0.01	2.99 ± 0.02	3.02 ± 0.02
SxPH-F	3.40 ± 0.03	3.26 ± 0.03	3.27 ± 0.03	3.07 ± 0.02	3.01 ± 0.02	2.99 ± 0.01	3.00 ± 0.01	2.99 ± 0.01
SxPH-R	3.41 ± 0.04	3.29 ± 0.05	3.26 ± 0.04	3.13 ± 0.02	3.00 ± 0.02	2.98 ± 0.02	2.96 ± 0.02	2.96 ± 0.02
Significance								
Irrigation	***	***	***	***	***	***	***	***
Biostimulant	ns	ns	ns	**	ns	ns	ns	ns
IrrigationxBiostimulant	ns	ns	ns	ns	ns	ns	ns	ns
